# Supplementary material for: Interaction of septin 7 and DOCK8 in equine lymphocytes reveals novel insights into signaling pathways associated with autoimmunity
Source: Sci Rep. 2018 Aug 17;8:12332. doi: 10.1038/s41598-018-30753-7 (PMC6098150; doi:10.1038/s41598-018-30753-7)
Supplement: Supplementary file 1 — Supplementary Information [file 41598_2018_30753_MOESM1_ESM.pdf]

Supplementary Information

Interaction of septin 7 and DOCK8 in equine lymphocytes reveals novel insights into signaling pathways associated with autoimmunity

Melanie Schauer[1], Kristina J H Kleinwort[1], Roxane L Degroote[1], Carmen Wiedemann[1], Elisabeth Kremmer[2], Stefanie M Hauck[3], Cornelia A Deeg[1*]

[1] Chair for Animal Physiology, Department of Veterinary Sciences, LMU Munich, 80539 Munich, Germany

[2] Institute for Molecular Immunology, Helmholtz Center Munich, German Research Center for Environmental Health GmbH, 81377 Munich, Germany

[3] Research Unit Protein Science, Helmholtz Center Munich, German Research Center for Environmental Health GmbH, 80939 Munich, Germany

*Corresponding author:

Cornelia A. Deeg, Chair for Animal Physiology, Department of Veterinary Sciences, LMU Munich, Veterinärstr. 13, D-80539 Munich. E-mail: Cornelia.Deeg@lmu.de,

Phone: + 49 (0)89–21802551

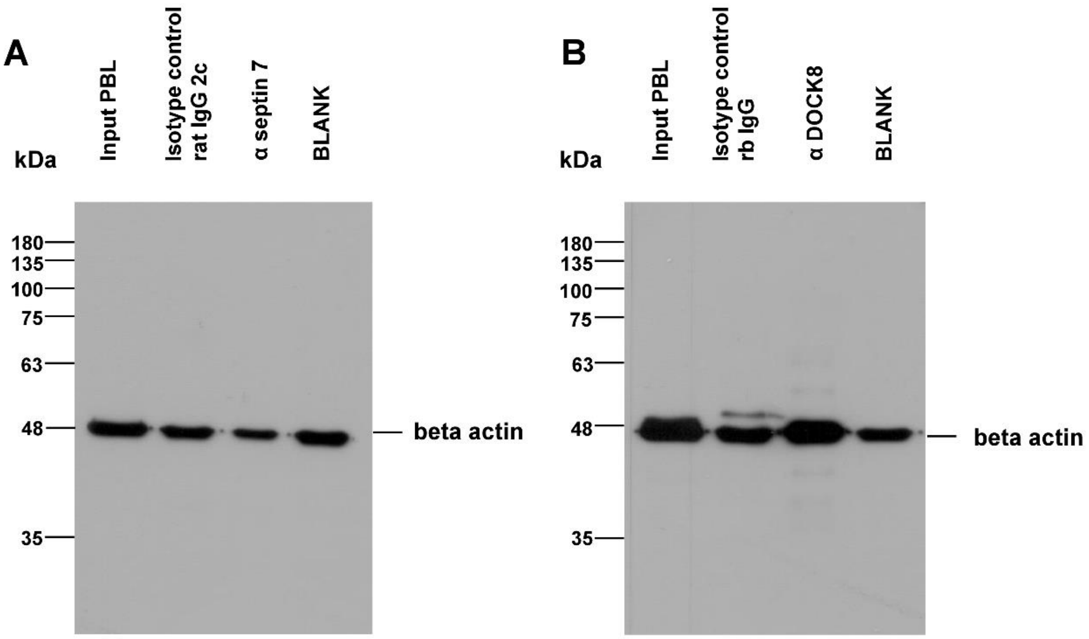

Supplementary Figure S1:

Original full-length blots of Panel A + B in Figure 1. (A) Beta actin signal abundance of PBL-lysate, isotype control (rat IgG 2c), blank and immunoprecipitate of septin 7. **(B)** Beta actin signal abundance of PBL-lysate, isotype control (rabbit IgG), blank and immunoprecipitate of DOCK8.

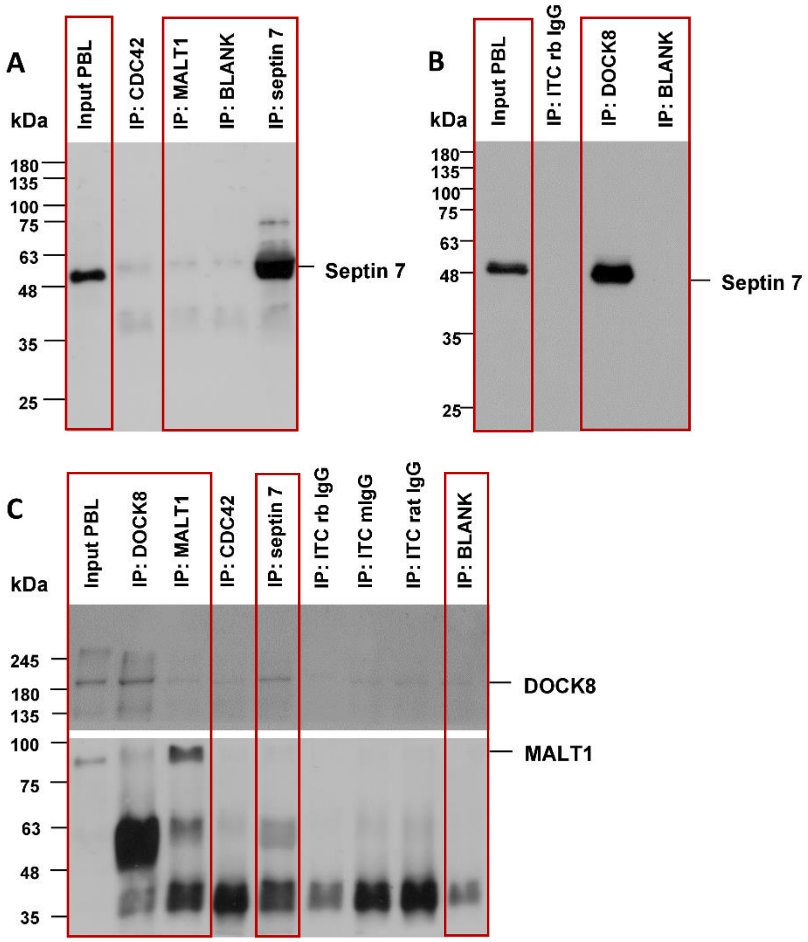

Supplementary Figure S2:

Grouped original full-length blots of Panel C in Figure 1 (marked lanes are shown in Figure 1, Panel C): (A + B) Septin 7 was detected in immunoprecipitates of DOCK8 (Panel B; IP: DOCK8) and septin 7 (Panel A; IP: septin 7) as well as in PBL-lysates (Panel A+B: Input PBL). Septin 7 was not precipitated by MALT1 (Panel A: IP: MALT1) or isotype control (Panel B: IP: ITC rb IgG). Immunoprecipitation of CDC42 did not work due to failure of antibody-binding. **(C)** Blot membrane was divided and incubated with polyclonal anti DOCK8 and polyclonal anti MALT1, raised in rabbit. DOCK8 was detected in immunoprecipitates of septin 7 (IP: septin7) and DOCK8 (IP: DOCK8) as well as in PBL-lysate (Input PBL). DOCK8 was not precipitated by MALT1 (IP: MALT1) or isotype controls (IP: ITC rb IgG, IP: ITC rat IgG). MALT1 was detected in immunoprecipitate of MALT1 (IP: MALT1) and in PBL-lysate (Input PBL), but not in immunoprecipitates of DOCK8 (IP: DOCK8), septin 7 (IP: septin 7) or respective isotype controls (IP: ITC rb IgG, IP: ITC rat IgG).

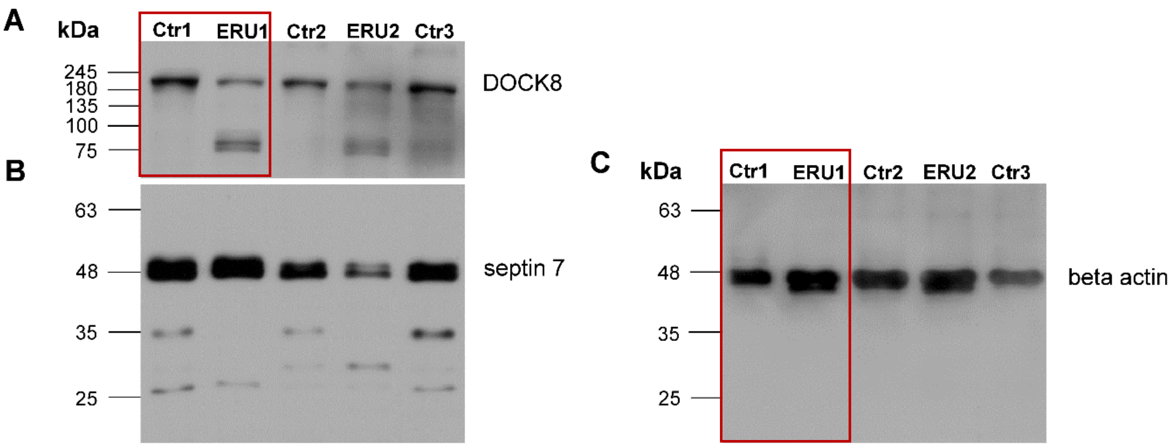

Supplementary Figure S3:

Grouped original full-length blot (A-C cropped from the same membrane) of Figure 3 (marked lanes are shown in Figure 3A). Blot membrane was divided (A+B) and incubated with different antibodies. All abundances were normalized to respective beta actin signals (C) on the same membrane. **(A)** Signal abundances of DOCK8 in PBL of controls and ERU detected by western blot. **(B)** Signal abundances of septin 7 on same membrane. **(C)** Beta actin signal abundance.

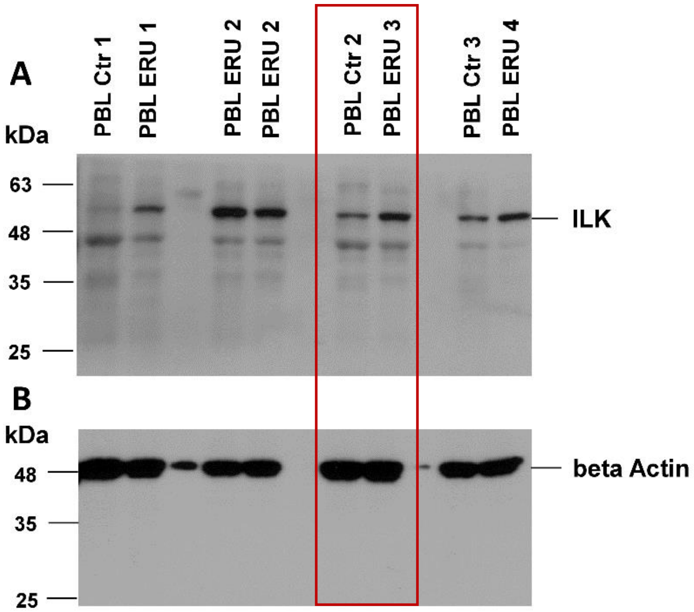

Supplementary Figure S4:

Grouped original full-length blot (A+B on same membrane) of Figure 5A (marked lanes are shown in Figure 5A). (A) Signal abundances of ILK in PBL of controls and ERU detected by western blot. **(B)** All abundances were normalized to beta actin signal abundance on the same membrane.

	**Present study**	***Tokhtaeva et al., 2015***	***Schneider et al., 2013***	***Nakahira et al., 2010***	***Spiliotis et al., 2005***	***Kinoshita et al., 2002***
**Bait protein**	Septin 7	Septin 2	CDc11p septin	Septins 1-10 (septin 7 interactors shown)	Septin 2, septin 6	Septin 2, septin 6, septin 7 (shown)
**Interacting Proteins (gene symbols)**	**ARPC2**, H2A, **CDC42**, CAPZA1, **MYL6, ACTB, SEP6,** HIST1H2BJ, VIM, FGA, CRP, **ACTN1, SEP7,** DOCK8, **MYO1G**, **FLOT2**, S100A8, TMOD3, CTSG, **SEP2**	**SEP7**, SEP11, **SEP6**, **SEP2**, SEP8, SEP3, SEP9, SEP5, SEP4, SEP10, CLTC, FLOT1, **FLOT2**, NSF, ARF1, DMN1, AP2A2, AP3B2, AP2S1, STXBP1, SYNJ1, SYN2, VAPB, SYNGR3, SYP, SYT1, Chaperons	**MYO1,** BNI5P	**SEP6**, SEP9, SEP4, SEP1, SEP10, SEP11, RALBP1, ANKRD12, ZNF451, CENP	**Actin cytoskeleton,** Phospholipid membrane,	**Actin Cytoskeleton**
**Model**	Horse	Mouse	Yeast	Human	HeLa cells, MDCK cells	Human
**Method**	Co-IP; LC-MS/MS	Co-IP; nLC-MS/MS	Split-UB, Pull-down assay	Yeast two-hybrid	Immunofluorescence	Co-sedimentation assay, immunofluorescence

Supplementary Table 1: Comparison of present study with known septin interactors from published studies
